# The characteristics of discharge prescriptions including pro re nata psychotropic medications for patients with schizophrenia and major depressive disorder from the survey of the “Effectiveness of guidelines for dissemination and education in psychiatric treatment (EGUIDE)” project

**DOI:** 10.1186/s12991-022-00429-8

**Published:** 2022-12-26

**Authors:** Yoshitaka Kyou, Norio Yasui-Furukori, Naomi Hasegawa, Kenta Ide, Kayo Ichihashi, Naoki Hashimoto, Hikaru Hori, Yoshihito Shimizu, Yayoi Imamura, Hiroyuki Muraoka, Hitoshi Iida, Kazutaka Ohi, Yuka Yasuda, Kazuyoshi Ogasawara, Shusuke Numata, Jun-ichi Iga, Takashi Tsuboi, Shinichiro Ochi, Fumitoshi Kodaka, Ryuji Furihata, Toshiaki Onitsuka, Manabu Makinodan, Hiroshi Komatsu, Masahiro Takeshima, Chika Kubota, Akitoyo Hishimoto, Kiyokazu Atake, Hirotaka Yamagata, Mikio Kido, Tatsuya Nagasawa, Masahide Usami, Taishiro Kishimoto, Saya Kikuchi, Junya Matsumoto, Kenichiro Miura, Hisashi Yamada, Koichiro Watanabe, Ken Inada, Ryota Hahimoto

**Affiliations:** 1 Department of Psychiatry, School of Medicine, Kitasato University, 1-15-1 Kitasato, Minami-Ku, Sagamihara, Kanagawa 252-0374 Japan; 2 Department of Psychiatry, Dokkyo Medical University School of Medicine, 880 Kitakobayashi, Shimotsuga, Mibu, Tochigi 321-0293 Japan; 3 Department of Pathology of Mental Diseases, National Institute of Mental Health, National Center of Neurology and Psychiatry, 4-1-1 Ogawahigashi, Kodaira, Tokyo 187-8553 Japan; 4 Department of Hospital Pharmacy, Hospital of University of Occupational and Environmental Health, 1-1 Iseigaoka, Yahatanishi-Ku, Kitakyushu-Shi, Fukuoka 807-8555 Japan; 5 Department of Neuropsychiatry, University of Tokyo Hospital, 7-3-1 Hongo, Tokyo, 113-8655 Japan; 6 Department of Psychiatry, Hokkaido University Graduate School of Medicine, Kita 15, Nishi 7, Kita-Ku, Sapporo, 060-8638 Japan; 7 Department of Psychiatry, Faculty of Medicine, Fukuoka University, 7-45-1 Nanakuma, Jyonan-Ku, Fukuoka City, Fukuoka 814-0180 Japan; 8 Department of Pharmacy, Kanazawa Medical University Hospital, 1-1 Daigaku, Uchinada, Kahoku, Ishikawa 920-0293 Japan; 9 Department of Neuropsychiatry, Kyorin University School of Medicine, 6-20-2 Shinkawa, Mitaka-Shi, Tokyo 181-8611 Japan; 10 Department of Psychiatry, Gifu University Graduate School of Medicine, 1-1 Yanagido, Gifu, Gifu 501-1194 Japan; 11 Life Grow Brilliant Mental Clinic, Medical Corporation Foster, 1-3-11 Oyodominami, Kitaku, Osaka 531-0075 Japan; 12 Center for Postgraduate Clinical Training and Career Development, Nagoya University Hospital, 65 Tsurumai-Cho, Showa-Ku, Nagoya, 466-8550 Japan; 13 Department of Psychiatry, Graduate School of Biomedical Science, Tokushima University, 3-8-15 Kuramoto-Cho, Tokushima, 770-8503 Japan; 14 Department of Neuropsychiatry, Ehime University Graduate School of Medicine, Shitsukawa, Toon, Ehime 791-0295 Japan; 15 Department of Neuropsychiatry, Molecules and Function, Ehime University Graduate School of Medicine, Shitsukawa, Toon, Ehime 791-0295 Japan; 16 Department of Psychiatry, The Jikei University School of Medicine, 3-25-8, Nishi-Shimbashi, Minatoku, Japan; 17 Agency for Health, Safety and Environment, Kyoto University, Yoshida Honmachi, Sakyo-Ku, Kyoto, 606-8501 Japan; 18 Department of Neuroimaging Psychiatry, Graduate School of Medical Sciences, Kyushu University, 3-1-1, Maidashi, Higashiku, Fukuoka, 812-8582 Japan; 19 Department of Psychiatry, Nara Medical University School of Medicine, 840 Shijocho Kashihara, Nara, 634-8522 Japan; 20 Department of Psychiatry, Tohoku University Hospital, 1-1 Seiryo-Machi, Aobaku, Sendai 980-8573 Japan; 21 Department of Neuropsychiatry, Akita University Graduate School of Medicine, 1-1-1, Hondo, Akita City, Akita 010-8543 Japan; 22 Department of Psychiatry, National Center Hospital, National Center of Neurology and Psychiatry, 4-1-1- Ogawahigashi, Kodaira, Tokyo 187-8553 Japan; 23 Department of Psychiatry, Yokohama City University Graduate School of Medicine, 3-9 Fukuura, Kanazawa, Yokohama, 236-0004 Japan; 24 Health Administration Center (Kyusyu Region), Nippon Telegraph and Telephone West Corporation, 13-8 DOIMACHI Bld.2F, Kamikawabatamachi, Hakata-Ku, Fukuoka, 812-0026 Japan; 25 Division of Neuropsychiatry, Department of Neuroscience, Yamaguchi University Graduate School of Medicine, 1-1-1 Minami-Kogushi, Ube, Yamaguchi 755-8505 Japan; 26 Kido Clinic, 244 Hounoki, Imizu, Toyama 934-0053 Japan; 27 Department of Neuropsychiatry, Graduate School of Medicine and Pharmaceutical Sciences, University of Toyama, 2630 Sugitani, Toyama, 930-0194 Japan; 28 Department of NeuroPsychiatry, Kanazawa Medical University, 1-1 Daigaku, Uchinada-Machi, Ishikawa, 920-0293 Japan; 29 Department of Child and Adolescent Psychiatry, Kohnodai Hospital, National Center for Global Health and Medicine, 1-7-1 Kohnodai, Ichikawa, Chiba 272-8516 Japan; 30 Hills Joint Research Laboratory for Future Preventive Medicine and Wellness, Keio University School of Medicine, 35 Shinanomachi, Shinjuku-Ku, Tokyo 160-8582 Japan; 31 Department of Neuropsychiatry, Hyogo Medical University, 1-1, Mukogawa-Cho, Nishinomiya, Hyogo 663-8501 Japan

**Keywords:** Depression, EGUIDE, Pro re nata, Psychotropic, Schizophrenia

## Abstract

**Background:**

Several guidelines recommend monotherapy in pharmacotherapy for schizophrenia and major depressive disorder. The content of regular prescriptions has been reported in several studies, but not enough research has been conducted on the content of pharmacotherapy, including pro re nata (PRN) medications. The purpose of this study was to evaluate the content of pharmacotherapy, including PRN medications, and to clarify the relationship with regular prescriptions.

**Methods:**

We used data from the “Effectiveness of Guidelines for Dissemination And Education in psychiatric treatment” (EGUIDE) project to investigate the presence or absence of PRN psychotropic medications at discharge for each drug category. We compared the PRN psychotropic prescription ratio at discharge by diagnosis for each drug category. The antipsychotic monotherapy ratio and no prescription ratio of other psychotropics for schizophrenia at discharge and the antidepressant monotherapy ratio and no prescription ratio of other psychotropics for major depressive disorder at discharge were calculated for each regular prescription, including PRN psychotropic medications, as quality indicators (QIs). Spearman's rank correlation test was performed for QI values of regular prescriptions and the QI ratio between regular prescriptions and prescriptions including PRN medications for each diagnosis.

**Results:**

The PRN psychotropic prescription ratio at discharge was 28.7% for schizophrenia and 30.4% for major depressive disorder, with no significant differences by diagnosis. The prescription ratios of PRN antipsychotic medications and PRN antiparkinsonian medications were significantly higher for schizophrenia. The prescription ratios of PRN anxiolytic and hypnotic and PRN antidepressant medications were significantly higher for patients with major depressive disorder. For both schizophrenia and major depressive disorder, the QI was lower for discharge prescriptions, including PRN medications, than for regular prescriptions. QI values for regular prescriptions and the QI ratio were positively correlated.

**Conclusions:**

Considering PRN psychotropic medications, the monotherapy ratio and no prescription ratio of other psychotropics at discharge decreased in pharmacotherapy for schizophrenia and major depressive disorder. A higher ratio of monotherapy and no prescription of other psychotropics on regular prescriptions may result in less concomitant use of PRN psychotropic medications. Further studies are needed to optimize PRN psychotropic prescriptions.

**Supplementary Information:**

The online version contains supplementary material available at 10.1186/s12991-022-00429-8.

## Background

Schizophrenia and major depressive disorder are serious chronic disorders that cause impaired social functioning [1, 2], and pharmacotherapy is an important part of their treatment. Several guidelines recommend monotherapy with antipsychotics for schizophrenia [3, 4] and monotherapy with antidepressants for major depressive disorder [5–8]. The “Effectiveness of Guidelines for Dissemination and Education in psychiatric treatment” (EGUIDE) project is a nationwide multicenter study launched in Japan in 2016 [9]. The EGUIDE project is an educational program for psychiatrists on guidelines for schizophrenia and major depressive disorder, and the content of inpatient treatment is quantified as a quality indicator (QI) and evaluated over time [10–19]. A QI is an indicator for quality of care by assessing the structure, process, and result of medical care and helps to assess whether a patient's care is consistent with the evidence-based standard of care [20]. The results from the EGUIDE project show monotherapy rates at discharge and concomitant rates of other psychotropic medications as QIs of treatment [10, 11]. For example, the antipsychotic monotherapy ratio in the inpatient treatment of schizophrenia in Japan is approximately 60%, and the antidepressant monotherapy ratio in the inpatient treatment of major depressive disorder in Japan is approximately 60%. However, these QIs were calculated for regular prescriptions that are taken daily on a regular basis.

In the psychiatric field, pro re nata (PRN) psychotropic medications are often used “as needed” for psychiatric symptoms such as agitation and insomnia, in addition to regular pharmacotherapy. It has been reported that PRN psychotropic medications are prescribed to 70–90% of patients hospitalized for psychiatric disorders [21, 22]. Moreover, the study from the EGUIDE project reported that the PRN psychotropic prescription ratio was approximately 30% for inpatients with schizophrenia and major depressive disorder, even for discharge prescriptions [15]. Previous studies on the frequency of PRN psychotropic medication use have been limited to local settings, at a single or a few facilities, and to a wide range of psychiatric diagnoses. A study in an adolescent acute psychiatric ward reported that the average use of PRN psychotropic drugs was 0.35 times/day (standard deviation (SD) = 0.60) [23]. In addition, a single-center study on 205 patients admitted for the treatment of schizophrenia reported that the use of PRN psychotropic drugs was 0–4.18 times/day, with a mean of 0.48 times/day (SD = 0.73) [24]. These reports suggest that PRN psychotropic medication is a frequently used treatment. Therefore, QIs, such as the monotherapy ratio, should be evaluated not only for regular prescriptions but also for pharmacotherapies, including PRN psychotropic medications. There is no high-quality evidence on the effectiveness of PRN psychotropic medications, and PRN psychotropic drug use is based on clinical experience and habits [25]. Furthermore, it has been suggested that PRN psychotropic drug prescriptions are at risk of leading to polypharmacy and high-dose prescribing [15, 26, 27]. It has also been suggested that the use of PRN psychotropic medications may in fact prolong hospital stay and increase readmission rate [24]. Thus, PRN psychotropic medications are widely used without sufficient evidence to recommend their use, and factors related to their prescription need to be clarified.

In this study, we aimed to reevaluate the pharmacotherapy of inpatients with schizophrenia and major depressive disorder at discharge in Japan, including the use of PRN psychotropic drugs. The data from the EGUIDE project were used to examine the content of prescriptions that included PRN psychotropic drugs for each drug category.

## Methods

This study was a continuous, nationwide, cross-sectional study. This study was approved by the ethics committees of the National Center for Neuropsychiatry and Neurology (approval number B2022-004) and the participating EGUIDE sites. This study was conducted in compliance with the Declaration of Helsinki and its amendments. The study protocol was registered in the University Hospital Medical Information Network Registry (UMIN000022645). We created a dataset of the treatment at discharge between April and September of each year from 2016 to 2020 from the 240 hospitals that participated in the EGUIDE project. Using this dataset, a pharmacotherapy survey was conducted, including PRN psychotropic prescriptions, on patients with diagnoses of schizophrenia and major depressive disorder discharged from each facility between April and September of the first year of participation in the EGUIDE project. Patients were able to opt out of the purpose and procedures of the study and refuse study participation. We gathered the medical record information of patients at each institution with opt-out consent. Diagnoses of schizophrenia and major depressive disorder were made according to the Diagnostic and Statistical Manual of Mental Disorders 5th edition [28]. For patients who were hospitalized multiple times, only the treatment of the first hospitalization was included. Patients whose discharge prescriptions were unknown were excluded from the study. In addition, we excluded patients with schizophrenia who did not have regular prescriptions of antipsychotics at discharge and patients with major depressive disorder who did not have regular prescriptions of antidepressants at discharge. Prescription data of 2498 patients who were diagnosed with schizophrenia and 1022 patients who were diagnosed with major depressive disorder (MDD) were gathered from 97 institutions.

We investigated the age at discharge and sex of the patients and the presence or absence of PRN psychotropic prescriptions at discharge for each psychotropic drug category. We performed t-test and χ2 tests on the association between each diagnosis and age; and sex. We then performed χ^2^ tests on the association between each diagnosis and PRN psychotropic prescription ratio at discharge for each psychotropic drug category.

In addition, QI values for regular prescriptions at discharge and for prescriptions including PRN psychotropic drugs at discharge were calculated. Additional file 1: Tables S1 and S2 show the specifics of QIs in this study. Furthermore, the ratio of QI values for prescriptions, including PRN medications, to QI values for regular prescriptions was calculated. Spearman's rank correlation test was performed on QI values for regular prescriptions and the QI ratio between regular prescriptions and prescriptions including PRN medications for each diagnosis. Because we performed the analysis 7 times throughout this study, the significance level of 5% was set at 5.5 × 10^–3^ (0.05/9) with Bonferroni correction due to multiple testing. All statistical analyses were performed using IBM SPSS Statistics 26.0 (IBM Co., Armonk, NY, USA) and Excel (Microsoft, Redmond, WA, USA).

## Results

The PRN psychotropic prescription ratio at discharge for each drug category and demographic data for the patients are shown in Table 1. There was no difference in the PRN psychotropic prescription ratio between schizophrenia and MDD. For each psychotropic category, the PRN antipsychotic prescription ratio was significantly higher for schizophrenia than for MDD (*p* = 4.9 × 10^–9^, *df* (1) = 34.22). The PRN anxiolytic and hypnotic prescription ratio was significantly higher for MDD than for schizophrenia (*p* = 2.0 × 10^–3^, *df* (1) = 9.51). The PRN antidepressant prescription ratio was significantly higher for MDD than for schizophrenia (*p* = 9.4 × 10^–6^, *df* (1) = 19.63). The PRN antiparkinsonian prescription ratio was significantly higher for schizophrenia than for MDD (*p* = 1.5 × 10^–4^, *df* (1) = 14.42).

**Table 1:** Demographic and clinical data

	Schizophrenia (*n* = 2498)	Major depressive disorder (*n* = 1022)	*p*-value
Age (mean ± SD)	45.63 ± 15.60	58.65 ± 17.26	1.1 × 10^–98^
Sex (female, *n*, %)	1354 (54.2)	666 (65.2)	2.4 × 10–9
PRN psychotropic prescription ratio (*n*, %)	718 (28.7)	311 (30.4)	0.32
PRN antipsychotic prescription ratio (*n*, %)	443 (17.7)	101 (9.9)	*4.9 × 10^–9^
PRN anxiolytic and hypnotic prescription ratio (*n*, %)	465 (18.6)	237 (23.2)	*2.0 × 10^–3^
PRN antidepressant prescription ratio (*n*, %)	13 (0.5)	22 (2.2)	*9.4 × 10^–6^
PRN antiparkinsonian prescription ratio (*n*, %)	55 (2.2)	4 (0.4)	*1.5 × 10^–4^

As the level of significance, *p* < 5.5 × 10–3, was within the 5% significance level, based on the Bonferroni correction, it was considered in the multiplicity of the tests

*PRN* pro re nata, *SD* standard deviation

^*^p < 0.05, after the Bonferroni correction

The PRN prescription ratios by drug category for patients with PRN psychotropic prescriptions at discharge are shown in Additional file 1: Table S3. Among patients with schizophrenia, more than half had PRN antipsychotic prescriptions and PRN anxiolytic and hypnotic prescriptions, whereas less than 10% had PRN antidepressant prescriptions and PRN antiparkinsonian prescriptions. Among patients with MDD, more than half had PRN anxiolytic and hypnotic prescriptions, whereas less than 10% had PRN antidepressant prescriptions and PRN antiparkinsonian prescriptions.

In addition, Tables 2 and 3 show the QI values for regular prescriptions, prescriptions including PRN medications, and the QI ratio between regular prescriptions and prescriptions including PRN medications.

**Table 2:** QI values for patients with schizophrenia

QI (*n* = 2498)	QI values for regular prescriptions	QI values for prescriptions including PRN	QI ratio between regular prescriptions and prescriptions including PRN
Antipsychotics monotherapy ratio (*n*, %)	1404 (56.2)	1266 (50.7)	0.902
Antipsychotics monotherapy ratio without any other psychotropics (*n*, %)	429 (17.2)	344 (13.8)	0.802
No prescription ratio of anxiolytics and hypnotics (*n*, %)	847 (33.9)	727 (29.1)	0.858
No prescription ratio of antidepressants (*n*, %)	2295 (91.9)	2292 (91.8)	0.999
No prescription ratio of mood stabilizers and antiepileptics (*n*, %)	1857 (74.3)	1857 (74.3)	1.00
No prescription ratio of antiparkinsonian drugs (*n*, %)	1755 (70.3)	1732 (69.3)	0.987

*PRN* pro re nata, *QI* quality indicator

**Table 3:** QI values for patients with major depressive disorder

QI (*n* = 1022)	QI values for regular prescriptions	QI values for prescriptions including PRN	QI ratio between regular prescriptions and prescriptions including PRN
Antidepressant monotherapy ratio (*n*, %)	774 (75.7)	766 (75.0)	0.990
Antidepressant monotherapy ratio without any other psychotropics (*n*, %)	97 (9.5)	76 (7.4)	0.784
No prescription ratio of anxiolytics and hypnotics (*n*, %)	251 (24.6)	218 (21.3)	0.869
No prescription ratio of antipsychotics (*n*, %)	516 (50.5)	482 (47.2)	0.934
No prescription ratio of mood stabilizers and antiepileptics (*n*, %)	891 (87.2)	891 (87.2)	1.00
No prescription ratio of antiparkinsonian drugs (*n*, %)	999 (96.8)	985 (96.4)	0.996

*PRN* pro re nata, *QI* quality indicator

**Fig. 1:**
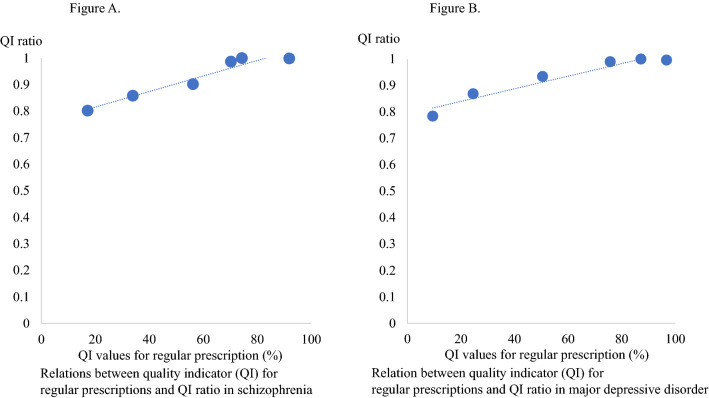
Relations between quality indicator (QI) for regular prescriptions and QI ratio in schizophrenia in Figure **A** (left-hand) and in major depressive disorder in Figure **B** (right-hand)

A positive correlation was found between QI values for regular prescriptions and the QI ratio for schizophrenia [r_s_ = 0.965, *p* = 1.8 × 10^–3^] (Fig. 1A). A positive correlation was also found between QI values for regular prescriptions and the QI ratio for MDD [r_s_ = 0.963, *p* = 2.0 × 10^–3^)] (Fig. 1B).

## Discussion

This is the first study to investigate PRN psychotropic prescriptions at discharge in inpatient treatment of schizophrenia and MDD by drug category. PRN psychotropic medications were prescribed at discharge to 28.7% of patients with schizophrenia and 30.4% of patients with MDD. There were no significant differences in the PRN psychotropic prescription ratio by diagnosis. On the other hand, the PRN antipsychotic prescription ratio and PRN antiparkinsonian prescription ratio were significantly higher for patients with schizophrenia than for those with MDD. The PRN anxiolytic and hypnotic prescription ratio and PRN antidepressant prescription ratio were significantly higher for patients with MDD than for patients with schizophrenia. This difference suggests that PRN psychotropic medications in certain drug categories are more likely to be prescribed depending on the diagnosis. Generally, antipsychotics are prescribed for SZ and antidepressants for MDD [3–8]. Therefore, it is obvious that the kind of prescribed medication depends on the diagnosis. It has been reported that PRN antipsychotic medications aimed at calming acute agitation are more common for patients with schizophrenia [26]. For this reason, PRN antipsychotic prescriptions might be more common for patients with schizophrenia than for those with MDD. PRN antidepressant medications were prescribed at low rates for both schizophrenia and MDD, possibly because antidepressants have fewer sedative effects [29, 30] and take time to develop their effects [31]. In addition, the guidelines for patients with SZ do not recommend prescribing antidepressants for SZ [3, 4]. Nevertheless, PRN antidepressants were prescribed to some SZ patients in this study. Therefore, education and dissemination of guideline-based treatment is required for PRNs as well. Since anticholinergics are widely used for the treatment and prevention of extrapyramidal symptoms produced by antipsychotics, especially acute dystonia [32], the PRN antiparkinsonian prescription ratio may have been higher for schizophrenia than for MDD. Insomnia is a frequent symptom in both schizophrenia and MDD [33–35] and may be one reason for the high PRN anxiolytic and hypnotic prescription ratio for both disorders.

In this study, the QI values decreased with the inclusion of PRN prescriptions for all QIs except no prescription ratio of mood stabilizers and antiepileptics. A previous study suggested that the polypharmacy ratio increases when PRN prescriptions are included [36], and a similar trend might be observed in this study. Furthermore, a positive correlation was found between QI values for regular prescriptions and the QI ratio for both schizophrenia and MDD. This means that higher QI values for regular prescriptions indicate a smaller reduction in QI values for prescriptions including PRN medications. Higher QI values for regular prescriptions indicate that there are fewer concomitant medications in that drug category on regular prescriptions, and a smaller reduction in QI values for prescriptions including PRN medications indicates fewer PRN prescriptions in that drug category. In other words, these results suggest that drug categories that are less likely to be used in combination on regular prescriptions are less likely to be prescribed as PRN medications. Despite several guidelines [3–8] recommending monotherapy for the treatment of schizophrenia and MDD, in clinical practice, both schizophrenia and MDD are treated with multiple psychotropics [37–39]. PRN psychotropic prescriptions are habitually practiced without sufficient evidence [25], and the need for guidelines on the proper use of PRN medications has been pointed out [24, 40, 41]. The results of this study suggest that promoting monotherapy as recommended in the guidelines, taking PRN medications into consideration, may lead to a reduction in PRN psychotropic prescriptions, which in turn may lead to an improvement in the QI value of each drug category. It has also been noted that PRN psychotropic medications for hospitalized patients may continue to be prescribed without regular monitoring of their use, and the need for education of health care providers regarding PRN psychotropic medications has been noted [24]. The EGUIDE project reported that educational programs on guidelines improved the clinical knowledge of participants [9], and there is a need to accumulate evidence on PRN psychotropic medications and provide appropriate education regarding PRN medications in the future.

## Limitations

There are several limitations in this study. First, this study did not assess the severity of psychiatric symptoms using a rating scale such as the Positive and Negative Syndrome Scale or the Hamilton Depression Rating Scale. Since treatment for patients with SZ and MDD may be influenced by the severity of the patients [3–8], further studies are needed taking into account the severity of illness. Second, this study examined only the presence or absence of PRN psychotropic prescriptions at discharge and did not assess the frequency of PRN medication use during hospitalization or the amount used per visit. Therefore, further studies are needed taking into account the frequency of PRN use during hospitalization and the amount of PRN used per visit. Third, the data in this study were collected from medical institutions that voluntarily participated in the survey, approximately half of which were university hospitals, which limits the generalizability of the results.

## Conclusion

Regarding PRN psychotropic medications, the monotherapy ratio and no prescription of other psychotropic medications ratio at discharge decreased in the pharmacotherapy for schizophrenia and MDD. A higher ratio of monotherapy and no prescription of other psychotropics on regular prescriptions may result in less concomitant use of PRN psychotropic medications. Further studies are needed to optimize PRN psychotropic prescriptions.

## Supplementary Information

**Additional file 1: Table S1.** Definition of Quality Indicators for patients with schizophrenia in this study. **Table S2.** Definition of Quality Indicators for patients with major depressive disorder in this study. **Table S3.** PRN prescription ratio at discharge by drug category.

## Data Availability

The datasets generated and/or analyzed during the current study are not publicly available due to privacy and ethical restrictions (i.e., we did not obtain informed consent on the public availability of raw data) but are available from the corresponding author on reasonable request.

## Abbreviations

df: Degrees of freedom
EGUIDE: The effectiveness of guidelines for dissemination and education in psychiatric treatment
MDD: Major depressive disorder
PRN: Pro re nata
QI: Quality indicator
